# The Differential Effect of *NAT2* Variant Alleles Permits Refinement in Phenotype Inference and Identifies a Very Slow Acetylation Genotype

**DOI:** 10.1371/journal.pone.0044629

**Published:** 2012-09-06

**Authors:** Jhon D. Ruiz, Carmen Martínez, Kristin Anderson, Myron Gross, Nicholas P. Lang, Elena García-Martín, José A. G. Agúndez

**Affiliations:** 1 Department of Pharmacology, Medical School, University of Extremadura, Badajoz & Cáceres, Spain; 2 Division of Epidemiology & Community Health, School of Public Health, University of Minnesota, Minneapolis, Minnesota, United States of America; 3 Department of Laboratory Medicine and Pathology, School of Medicine, University of Minnesota, Minneapolis, Minnesota, United States of America; 4 Department of Surgery, University of Arkansas Medical Sciences, Little Rock, Arkansas, United States of America; 5 Department of Biochemistry & Molecular Biology, School of Biological Sciences, University of Extremadura, Cáceres, Spain; I2MC INSERM UMR U1048, France

## Abstract

Indirect evidences suggest that acetylation phenotype categories are heterogeneous and that subcategories, related to specific *NAT2* variant alleles might exist. We analyzed the *in vivo* acetylation phenotype and genotype in 504 north-American subjects of Caucasian origin. The analyses of the SNPs rs1801280 and rs1799930 allowed the discrimination of five categories with different acetylation status within the study population. These categories are related to the distinct effect of *NAT2* alleles on the acetylation status *in vivo* and to the occurrence of a gene-dose effect. These five phenotype categories, from higher to lower acetylation capacity, correspond to the genotypes NAT2*4/*4, NAT2*4/*5 or *4/*6, NAT2*5/*5, NAT2*5/*6 and NAT2*6/*6 (p≤0.001 for all comparisons). The NAT2*6/*6 genotype correspond to a phenotype category of very-slow acetylators. The refinement in phenotype prediction may help to identify risks associated to phenotype subcategories, and warrants the re-analysis of previous studies that may have overlooked phenotype subcategory-specific risks.

## Introduction

The widespread use of genetic biomarkers as surrogate endpoints aiming to describe risks, exposures, intermediate effects of treatments, and biologic mechanisms is a goal that scientists have long been pursuing. The adoption of any genetic test as a surrogate biomarker requires previous demonstration of its analytical and clinical validity as well as its clinical utility, and increasing the predictive capacity of genetic biomarkers is one of the major problems that we have to solve in order to transfer advances in pharmacogenomics to routine clinical practice. Determination of the polymorphic acetylation (*NAT2* genotype or phenotype) was initially proposed to predict adverse reactions in patients with tuberculosis receiving isoniazid, prior to the concomitant administration of procainamide and phenytoin, and to analyze the role of NAT2 in drug interactions. These effects, together with the role of *NAT2* in cancer risk, in non-malignant spontaneous disorders and in drug response and toxicity, make *NAT2* a relevant target for pharmacogenomic testing in clinical practice [1], [2].

Nearly fifty years ago, Evans et al. demonstrated that acetylation of isoniazid was bimodally distributed and that the *in vivo* acetylation status was inheritable [3], [4]. Since then, traditional phenotype determination by inference from genetic analyses has classified the population in three groups: rapid, intermediate and slow acetylators. Although this classification of individuals into three phenotype categories is widely accepted, it would be desirable to refine further the predictive capacity of acetylation pharmacogenomic testing [5]. Heterologous expression of NAT2 allozymes provided indirect evidence suggesting a differential effect of *NAT2* variant alleles and hence heterogeneity within the slow acetylation phenotype (reviewed in [6]).

This study aims to analyze whether this evidence of heterogeneity within rapid and slow acetylators exists *in vivo*, whether commonly used pharmacogenomic tests are adequate for the inference of phenotype subcategories, and to measure the activities for such phenotype subcategories. Because acetyl metabolites may be pharmacologically active, or function as intermediates in toxic metabolic pathways, further refinement in phenotype prediction may help to identify risks associated to one or more of such phenotype subcategories.

## Methods

The subjects were drawn from a study previously described [7], [8], [9]. Briefly, cases (n = 93), of newly diagnosed cancer of the exocrine pancreas were recruited from all hospitals in the 7-county metropolitan area of the Twin Cities, Minnesota and the Mayo Clinic (from the latter, only cases residing in the Upper Midwest of the US were recruited). Controls (n = 411) were randomly selected from the general population and frequency matched to cases by age and sex (Table 1). All were Caucasian. Each participant provided written, informed consent prior to interview and blood draw. The study was approved by the Institutional Review Boards of the University of Minnesota and The Mayo Clinic, USA and by the Ethics Committee of the University of Extremadura, Spain.

**Table 1:** Characteristics of the individuals included in the study.

	Overall study group	Overall study group	Individuals selected	Individuals selected
	Male (n = 312)	Female (n = 192)	Male (n = 274)	Female (n = 161)
Age years (mean ± SD)	65.3±11.3	65.7±13.0	65.6±11.2	66.0±12.4
Never smokers (n; %)	104 (33.3%)	113 (58.9%)	89 (32.5%)	98 (60.1%)
Past smokers (n; %)	167 (53.5%)	59 (30.7%)	147 (53.6%)	47 (28.8%)
Current smokers (n; %)	41 (13.1%)	20 (10.4%)	38 (13.9%)	18 (11.0%)
Pack-years (mean ± SD)	37.4±30.6	23.7±21.2	37.8±30.7	23.2±19.9
Non-drinkers/drinkers	112/200	95/97	96/178	80/83
Servings of alcohol per week(mean ± SD)	9.1±11.2	4.9±5.2	9.4±11.6	5.2±5.4
Cases/Controls (n)	63/249	30/162	53/221	20/141

Individuals selected for phenotype inference refinement correspond to 435 individuals with genotypes NAT2*4/*4, *4/*5, *4/*6, *5/*5, *5/*6 or *6/*6 and phenotype/genotype concordance.

Pack-years calculation includes smokers and ex-smokers. Servings of alcohol per week include drinkers only.

In vivo NAT2 activity was measured with a widely used caffeine-based assay, as described by Butler et al. [10] with minor modifications as described elsewhere [8], [11]. The caffeine assay is highly accurate and reproducible, and it is considered as a gold standard for acetylation phenotyping. Details on accuracy and reproducibility were published elsewhere [10], [12], [13], [14], [15]. In brief, subjects ingested 200 mg of caffeine, following an overnight fast. Subjects refrained from the consumption of caffeine- and methylxanthine-containing foods and beverages from midnight until 5 h after the dose of caffeine. A urine specimen was collected 5 h after the administration of caffeine and samples were acidified and stored as described elsewhere [11]. Regarding HPLC analysis, 200 µl of urine were saturated with 125 mg of ammonium sulfate, and 6.0 ml of chloroform:isopropanol (95∶5) were added. Each sample was vortexed and centrifuged, and the organic phase was removed and evaporated to dryness. The residue was resuspended in 250 µl of 0.05% acetic acid, filtered, and frozen until analysis. Fifty µl of the extract were injected onto a Beckman C18 Ultrasphere octadecylsilane column (25 cm in length, 4.6-mm diameter, 5-µm particle size) and eluted with a 0.05% acetic acid-methanol solvent (flow rate, 1.2 ml/min).

Acetylation phenotype was assigned on the basis of a molar AFMU/1X ratio, which served as quantitative determinant of acetylation capacity with a cut-off value = 0.66 (log AFMU/1X = −0.18) in agreement with previous studies [11].

*NAT2* genotyping aimed to identify the signature SNPs for alleles corresponding to the NAT2*5, NAT2*6, NAT2*7 and NAT2*14 clusters, that is, rs1801280 (I114T), rs1799930 (R197Q), rs1799931 (G286E) and rs1801279 (R64Q), respectively. Although several *NAT2* alleles have been described (for an updated list of *NAT2* alleles and haplotypes see the website http://louisville.edu/medschool/pharmacology/consensus-human-arylamine-n-acetyltransferase-gene-nomenclature/nat_pdf_files/Human_NAT2_alleles.pdf), the SNPs analyzed in this study identify the vast majority of slow *NAT2* variant allele clusters [16], [17]. Genotyping was carried out by the use of TaqMan®probes (details available in Table S1). For every SNP analyzed, twenty samples with heterozygous genotypes and up to twenty samples with homozygous genotypes (homozygous non-mutated and homozygous mutated when available), were determined as blind duplicates. In all samples with genotype/phenotype discordance (n = 32) the genotypes were confirmed by the use of PCR-based mutation-specific amplification as described elsewhere [8] or by direct sequencing of the amplified fragments. In all cases the genotypes fully corresponded to those obtained with TaqMan probes. Haplotype assignation and phenotype inference: All possible haplotypes combining the four SNPs analyzed were constructed and their frequencies were analyzed by using PHASE and the *NAT2* haplotype table described elsewhere [16]. Phenotype inference was carried out as described elsewhere [16]. Putative departures of Hardy-Weinberg Equilibrium were calculated by using the software Haploview 4.1. Continuous variables (acetylation ratios), expressed as mean (SD), were compared with the Student’ T test, and tests for trend were calculated with the Spearman’s rank correlation by using the statistical software SPSS 15.0 for Windows (SPSS Inc. Chicago, Illinois, USA). A *p* value <0.05 was considered significant. When multiple comparisons were made, adjustments for multiple comparisons were carried out according to Bonferroni’s procedure.

## Results

The SNP frequencies and the genotypes observed in the 504 participants are summarized in Table 2. The degree of phenotype/genotype concordance by using the traditional phenotype classification (i.e. rapid/slow phenotypes), where NAT2*4 containing genotypes are considered a rapid phenotype, and other genotypes a slow phenotype, was equal to 93.7%. We selected 435 individuals with genotypes NAT2*4/*4, *4/*5, *4/*6, *5/*5, *5/*6 and *6/*6 and phenotype/genotype concordance for further analyses. These corresponded to 73 cases and 362 control subjects. Carriers of variant alleles NAT2*14 were not included in the analyses, because these alleles were not present in the study population (Table 2). In addition, carriers of the variant alleles NAT2*7 were not included in the comparisons because these alleles were rare in the population study (Table 2).

**Table 2:** *NAT2* SNP frequencies observed in the present study.

*SNP identifier*	Amino Acid	No.	Observedfrequency (%)	Expectedfrequency (%)	Hardy Weingberg’s P
**rs1801280 (** NAT2*5 **)**					
**T/T**	114 Ile/Ile	162	32.14	31.64	
**T/C**	114 Ile/Thr	243	48.22	49.22	0.647
**C/C**	114 Thr/Thr	99	19.64	19.14	
**rs1799930 (** NAT2*6 **)**					
**G/G**	197 Arg/Arg	263	52.18	52.88	
**G/A**	197 Arg/Gln	207	41.07	39.68	0.430
**A/A**	197 Gln/Gln	34	6.75	7.44	
**rs1799931 (** NAT2*7 **)**					
**G/G**	286 Gly/Gly	464	92.06	91.84	
**G/A**	286 Gly/Glu	38	7.54	7.99	0.209
**A/A**	286 Glu/Glu	2	0.40	0.17	
**rs1801279 (** NAT2*14 **)**					
**G/G**	64 Arg/Arg	504	100.0	100.0	
**G/A**	64 Arg/Gln	0	000.0	00.0	(–.–)
**A/A**	64 Gln/Gln	0	000.0	00.0	

Expected frequencies are calculated from observed allele frequency.

Table 3 shows the acetylation capacity of the six genotype categories analyzed in the study. The individuals with the genotype categories NAT2*4/*5 and NAT2*4/*6 showed similar acetylation values. However, for the rest of individuals, each genotype category corresponded to a distinct phenotype category, with non-overlapping 95% confidence intervals for the activity, and in all cases the differences between these categories were statistically significant. This provides *in vivo* evidence that in the absence of NAT2*4 alleles, variant alleles NAT2*5 and NAT2*6 confer different acetylation capacity. In addition, a gene-dose for these variant alleles can be observed within the slow acetylator phenotype, as there is a statistically significant trend to slower acetylation capacity among individuals with the genotypes as follows: NAT2*5/*5>NAT2*5/*6>NAT2*6*6 (Spearman's rank correlation with the number of NAT2*6 alleles, (log AFMU/1X = −0.359); *P*<0.001). These findings were not influenced by the sex of participants, age, smoking status, pack-years, drinking status, servings of alcohol per week as stated by multivariable linear regression, or by the case-control status (Table 4, Table S2). The effect of NAT2*7 in vivo could not be elucidated because of the low allele frequency in the study population. We identified only two carriers of the alleles NAT2*7 in homozygosity, with metabolic ratios equal to −0.51 and −0.96. The mean value (−0.74), is close to the mean value for carriers of the NAT2*6/*6 genotypes, thus suggesting that the NAT2*7 alleles in homozygosity may confer a very slow acetylation phenotype; although due to the sample size the comparisons of the acetylation phenotype were not statistically significant. Table S3 includes details of the log AFMU/1X ratios of carriers of NAT2*7.

**Table 3:** Acetylation ratios (log AFMU/1X) in subjects with different *NAT2* genotypes.

Phenotype	Genotype	Number	Mean Ratio	SD	95% CI min	95% CI max
**Overall rapid**	NAT2*4/any	**197**	**0.209**	**0.155**	**0.182**	**0.226**
Rapid	NAT2*4/*4	36	0.327	0.169	0.270	0.385
Rapid-Intermediate	NAT2*4/*5	95	0.170	0.139	0.142	0.199
Rapid-Intermediate	NAT2*4/*6	66	0.186	0.141	0.151	0.220
**Overall Slow**	**Slow/Slow**	**238**	**−0.537**	**0.147**	−**0.556**	−**0.518**
Slow	NAT2*5/*5	91	**−**0.480	0.140	−0.509	−0.451
Slow	NAT2*5/*6	115	−0.551	0.131	−0.575	−0.527
Slow	NAT2*6/*6	32	−0.646	0.149	−0,698	−0,592
						
**T-test**	**Genotype**	NAT2*4/*5	NAT2*4/*6	NAT2*5/*5	NAT2*5/*6	NAT2*6/*6
	NAT2*4/*4	p<0.0001	p<0.0001	p<0.0001	p<0.0001	p<0.0001
	NAT2*4/*5		p = 0.574	p<0.0001	p<0.0001	p<0.0001
	NAT2*4/*6			p<0.0001	p<0.0001	p<0.0001
	NAT2*5/*5				p = 0.0002	p<0.0001
	NAT2*5/*6					p = 0.0005

The 435 individuals (73 cases and 362 control subjects) with genotypes NAT2*4/*4, *4/*5, *4/*6, *5/*5, *5/*6 and *6/*6 and phenotype/genotype concordance were included in the comparison.

According to multiple comparison adjustment of the 15 genotype pairs according Bonferroni’s procedure, a *P* value ≤0.0033 is considered as significant. Individual number for p values <0.0001 are rounded as “p<0.0001”.

**Table 4:** Effect of the case-control status on the Acetylation ratios (log AFMU/1X) in subjects with different *NAT2* genotypes.

Genotype	Status	Mean Log ratio (SD)	95% CI min	95% CI max	Inter-group comparison
NAT2*4/*4	Case (n = 7)	0.273 (0.181)	0.105	0.441	
	Control (n = 29)	0.341 (0.167)	0.277	0.404	*p* = 0.373
NAT2*4/*5	Case (n = 20)	0.157 (0.197)	0.065	0.249	
	Control (n = 75)	0.181 (0.117)	0.154	0.209	*p* = 0.605
NAT2*4/*6	Case (n = 8)	0.166 (0.116)	0.077	0.254	
	Control (n = 58)	0.197 (0.140)	0.159	0.235	*p* = 0.474
NAT2*5/*5	Case (n = 11)	−0.496 (0.134)	−0.405	−0.586	
	Control (n = 80)	−0.479 (0.141)	−0.447	−0.510	*p* = 0.705
NAT2*5/*6	Case (n = 20)	−0.595 (0.122)	−0.536	−0.653	
	Control (n = 95)	−0.543 (0.132)	−0.516	−0.569	*p* = 0.103
NAT2*6/*6	Case (n = 7)	−0.714 (0.087)	−0.633	−0.795	
	Control (n = 25)	−0.627 (0.158)	−0.563	−0.691	*p* = 0.173

## Discussion

Differential effects of acetylation status by different slow acetylation alleles have been suggested previously, but to our knowledge they have not been formally evaluated *in vivo*. Indirect evidence from *in vitro* studies and from clinical association studies suggest that *NAT*2 variant alleles produce different functional effects, implying heterogeneity within the “slow” acetylator phenotype [6]. Antituberculosis drug-induced hepatotoxicity risk is particularly high in carriers of the NAT2*6/*6 allele, thus suggesting that these individuals may constitute a subcategory of “very slow” acetylators [18], [19]. These and other clinical association studies (reviewed in [6]) suggest that the NAT2 slow acetylator phenotype is heterogeneous, and that multiple slow acetylator phenotypes exist [20]. However, no clear association between *NAT2* variant alleles and *in vivo* phenotype categories among slow acetylator individuals has been proved so far. Our findings indicate that the NAT2*6 allele cluster is related with the slowest acetylation capacity *in vivo* with a gene-dose effect, thus demonstrating the occurrence of a category of “very slow acetylators” with the genotype NAT2*6 in homozygosity. Because of the ethnic origin of the population study, we were unable to dissect the effect of the allele clusters NAT2*7 and NAT2*14; it should, however, be emphasized that these clusters are rare in caucasian populations [21] and that the allele frequencies observed in this study are consistent with those reported for other Caucasian individuals [21], [22].

The effect of *NAT2* variant alleles may vary by substrate or with substrate concentration [6]. For instance, it has been shown that the NAT2*7 allele cause a different effect in the N-acetyltransferase activity towards 2-aminofluorene and to sulfamethazine [23]. Therefore the findings obtained in this study should not be extrapolated to other *NAT2* substrates without confirmation with every specific substrate. Nevertheless, our findings *in vivo* agree with findings obtained *in vitro* which suggests that the protein level expressed by common *NAT2* alleles is NAT2*4>NAT2*5>NAT2*6 [6], thus suggesting that the differential effect of *NAT2* alleles observed with the probe drug caffeine is likely to be relevant to other NAT2 substrates.

The aims of this study are to refine the phenotype inference of *NAT2* genotyping and the identification of clinically relevant associations of the new genotype categories with cancer risk, differential treatment response or clinical outcome are beyond the aims of the study. Although this study included patients with cancer of the exocrine pancreas and control subjects, no association of *NAT2* genotype categories with pancreatic cancer risk was observed, in agreement with previous studies [24], [25].

The findings reported in this study show that acetylation capacity *in vivo* is related to different *NAT2* genotypes among slow acetylators, and indicate that variations in the acetylation *NAT2* status among slow acetylator individuals result from the co-dominant expression of the NAT2*5 and NAT2*6 alleles or haplotypes, whose diplotypes are related to distinct slow acetylation phenotypes. Additional studies are required to go further in the refinement in phenotype inference, particularly in other human populations with different *NAT2* allele frequencies. It may be argued that the difference in function between the variants NAT2*5 and NAT2*6, although statistically significant, is a minor difference compared to the function of any genotype containing at least one NAT2*4 allele and therefore that the clinical relevance of this difference may be limited. However, NAT2*6/*6 homozygotes show roughly a 30% reduction on enzyme activity as compared to NAT2*5/*5 homozygotes. For comparison, the reduction on enzyme activity between NAT2*4 heterozygotes (intermediate acetylators) and NAT2*4/*4 homozygotes (rapid acetylators) in this study is 28%. A 30% reduction in activity among individuals who have a very impaired acetylation capacity may have a higher clinical relevance than a comparable reduction among individuals who have a high acetylation capacity. These findings provide a novel framework for evaluating interactions between *NAT2* genotype and adverse drug reactions or cancer risk.

## Supporting Information

Table S1 **Details of the genotyping procedures used in the present study.**
(DOCX)

Table S2 **Comparison of the Acetylation ratios (log AFMU/1X) in healthy subjects with different** ***NAT2*** **genotypes.**
(DOCX)

Table S3 **Details of the acetylation ratios of individuals carrying** NAT2*7.
(DOCX)
