# Supplementary material for: The Differential Effect of NAT2 Variant Alleles Permits Refinement in Phenotype Inference and Identifies a Very Slow Acetylation Genotype
Source: PLoS One. 2012 Sep 6;7(9):e44629. doi: 10.1371/journal.pone.0044629 (PMC3435299; doi:10.1371/journal.pone.0044629)
Supplement: Table S1 — Details of the genotyping procedures used in the present study. (DOCX) [file pone.0044629.s001.docx]

**Table S1.** Details of the genotyping procedures used in the present study

| ***SNP identifier*** | **Consequence** | **NAT2 allele cluster** | **TaqMan Assay Id.** | **PCR conditions** | **Allelic discrimination** |
| --- | --- | --- | --- | --- | --- |
| rs1801280 (T/C) | I114T | *NAT2*5* | C___1204093_20 | 92ºC 15 sec  60ºC 90 sec  45 cycles | Fluorescent dyes as indicated by the manufacturer |
| rs1799930 (G/A) | R197Q | *NAT2*6* | C___1204091_10 | 92ºC 15 sec  60ºC 90 sec  45 cycles | Fluorescent dyes as indicated by the manufacturer |
| rs1799931 (G/A) | G286E | *NAT2*7* | C____572770_20 | 92ºC 15 sec  60ºC 90 sec  45 cycles | Fluorescent dyes as indicated by the manufacturer |
| rs1801279 (G/A) | R64Q | *NAT2*14* | C____572771_10 | 92ºC 15 sec  60ºC 90 sec  45 cycles | Fluorescent dyes as indicated by the manufacturer |

According to standard procedures, all alleles lacking these SNPs are classified as *NAT2*4* for this analysis.
