# Supplementary material for: The Differential Effect of NAT2 Variant Alleles Permits Refinement in Phenotype Inference and Identifies a Very Slow Acetylation Genotype
Source: PLoS One. 2012 Sep 6;7(9):e44629. doi: 10.1371/journal.pone.0044629 (PMC3435299; doi:10.1371/journal.pone.0044629)
Supplement: Table S2 — Comparison of the Acetylation ratios (log AFMU/1X) in healthy subjects with different NAT2 genotypes. (DOCX) [file pone.0044629.s002.docx]

**Table S2.** Comparison of the Acetylation ratios (log AFMU/1X) in healthy subjects with different *NAT2* genotypes .

Healthy individuals

| **T-test** | **Genotype** | ***NAT2*4/*5*** | ***NAT2*4/*6*** | ***NAT2*5/*5*** | ***NAT2*5/*6*** | ***NAT2*6/*6*** |
| --- | --- | --- | --- | --- | --- | --- |
|  | ***NAT2*4/*4*** | p < 0.0001 | p < 0.0001 | p < 0.0001 | p < 0.0001 | p < 0.0001 |
|  | ***NAT2*4/*5*** |  | p = 0.633 | p < 0.0001 | p < 0.0001 | p < 0.0001 |
|  | ***NAT2*4/*6*** |  |  | p < 0.0001 | p < 0.0001 | p < 0.0001 |
|  | ***NAT2*5/*5*** |  |  |  | p < 0.0001 | p < 0.0001 |
|  | ***NAT2*5/*6*** |  |  |  |  | p = 0.0062 |
