# Supplementary material for: The Differential Effect of NAT2 Variant Alleles Permits Refinement in Phenotype Inference and Identifies a Very Slow Acetylation Genotype
Source: PLoS One. 2012 Sep 6;7(9):e44629. doi: 10.1371/journal.pone.0044629 (PMC3435299; doi:10.1371/journal.pone.0044629)
Supplement: Table S3 — Details of the acetylation ratios of individuals carrying NAT2*7. (DOCX) [file pone.0044629.s003.docx]

**Table S3.** Details of the acetylation ratios of individuals carrying *NAT2*7.*

| Genotype | log AFMU/1X  Mean (SD) | Phenotype |
| --- | --- | --- |
| ***NAT2*4/*7***  ***N= 6*** | 0.151 (0.325) | Rapid |
| ***NAT2*5/*7***  ***N = 20*** | -0.228 (0.391) | Slow |
| ***NAT2*6/*7***  ***N = 12*** | -0.355 (0.457) | Slow |
| ***NAT2*7/*7***  ***N = 2*** | -0.740 (---) | Slow |
